# Supplementary material for: Geography Shapes the Population Genomics of Salmonella enterica Dublin
Source: Genome Biol Evol. 2019 Jul 22;11(8):2220–31. doi: 10.1093/gbe/evz158 (PMC6703130; doi:10.1093/gbe/evz158)
Supplement: evz158_Supplementary_Data [file evz158_supplementary_data.zip › Supporting information legends.docx]

**Supporting information legends**

**FIG S1** Population structure of *S.* Dublin identified using core kmer content with the software KSNP3. Leaves are colored respective to the region of isolation. The outer ring is colored respective to isolation source and the outer bars are scaled to date of isolation. The higher the bar the more recent the isolate was cultured. Strong geographical clustering is observed with clades corresponding to regions of isolation.

**FIG S2** Comparison of the ancestral state phylogeny with the maximum-likelihood phylogeny. Major clades are collapsed for comparison purposes. The five major clades are conserved in both phylogenomic methods.

**FIG S3** Maximum-likelihood tree showing the phylogeny of 880 *S.* Dublin and 161 S. Enteritidis. *S.* Dublin leaves are colored respective to the region of isolation and one *S.* Enteritidis (AM933172) that was used to root the *S.* Dublin phylogeny. *S.* Dublin forms a single clade away from *S.* Enteritidis. Geographical clades are seen in the *S.* Dublin clade.

Supplemental Table 1 - Metadata for 880 genomes used in the study.

SUPPLEMENTAL TABLE 2 – Detailed information on prophage regions identified.

**Supplemental Table 3** – Details of antimicrobial resistance genes in *S.* Dublin

Supplemental Table 4 – List of *S.* Dublin specific genes
