# Supplementary figures and images for: Geography Shapes the Population Genomics of Salmonella enterica Dublin
Source: Genome Biol Evol. 2019 Jul 22;11(8):2220–31. doi: 10.1093/gbe/evz158 (PMC6703130; doi:10.1093/gbe/evz158)

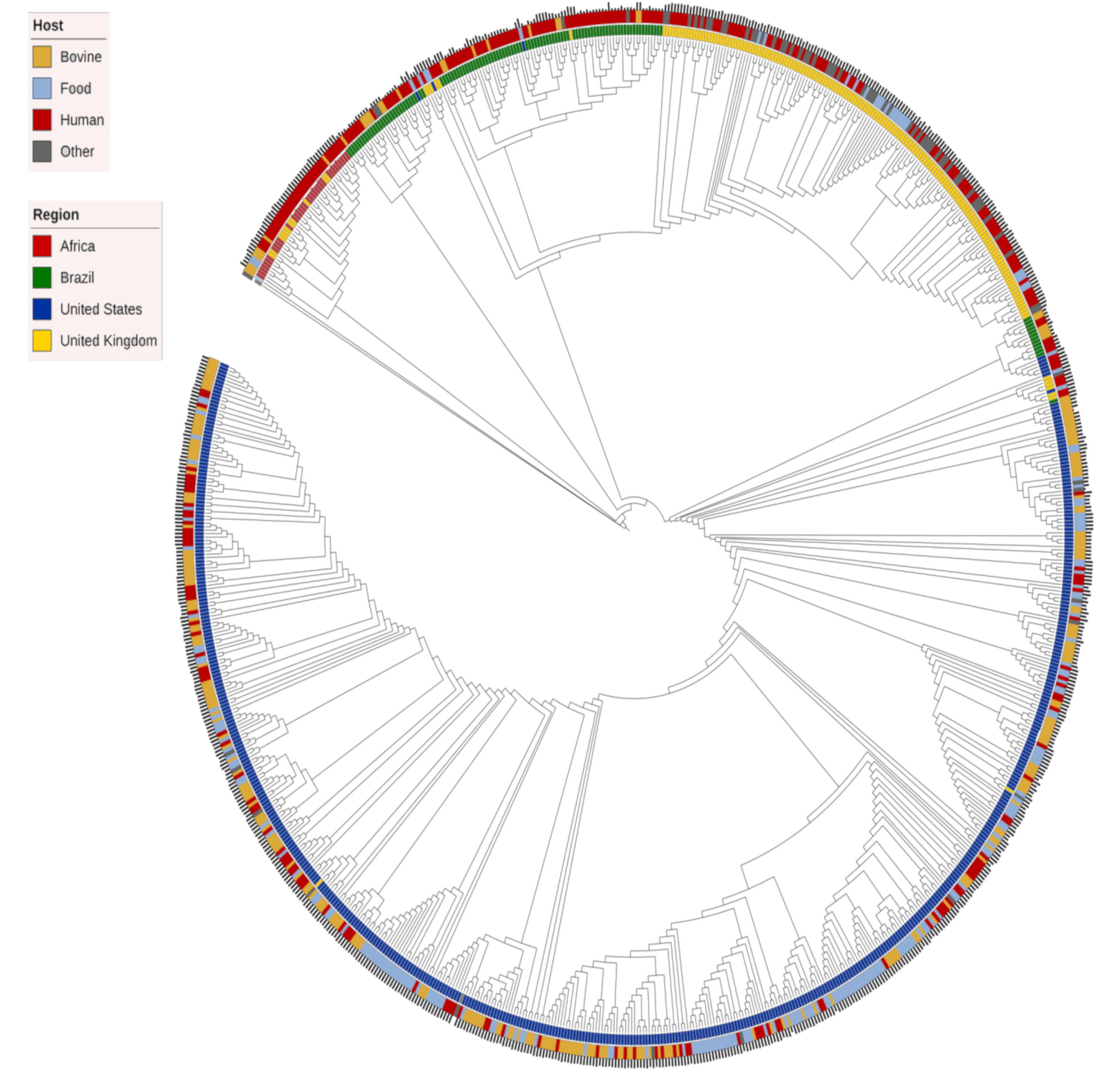

Supplement: evz158_Supplementary_Data [file evz158_supplementary_data.zip › Supplemental_FIG_01.tif]

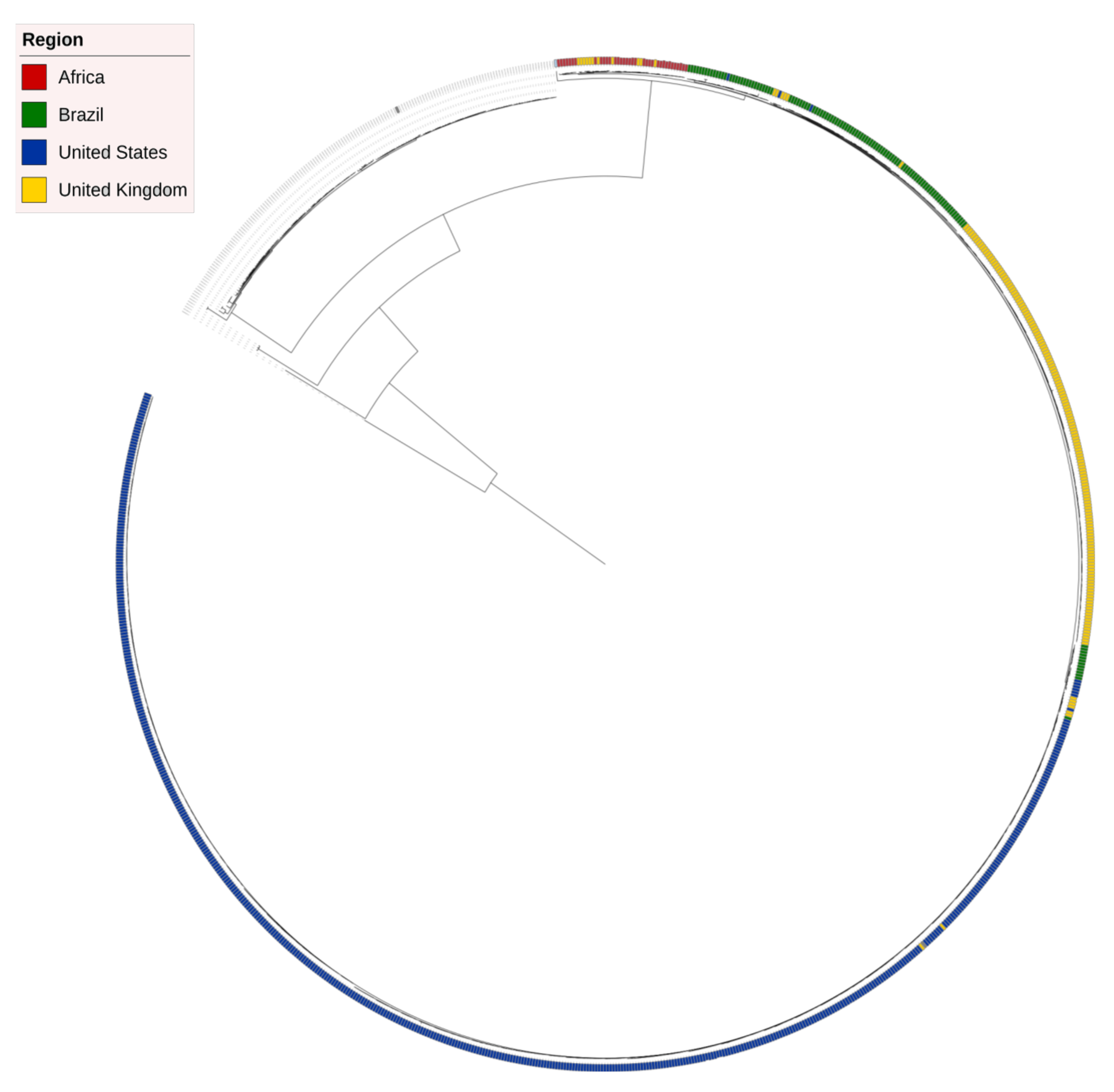

Supplement: evz158_Supplementary_Data [file evz158_supplementary_data.zip › Supplemental_FIG_02.tif]

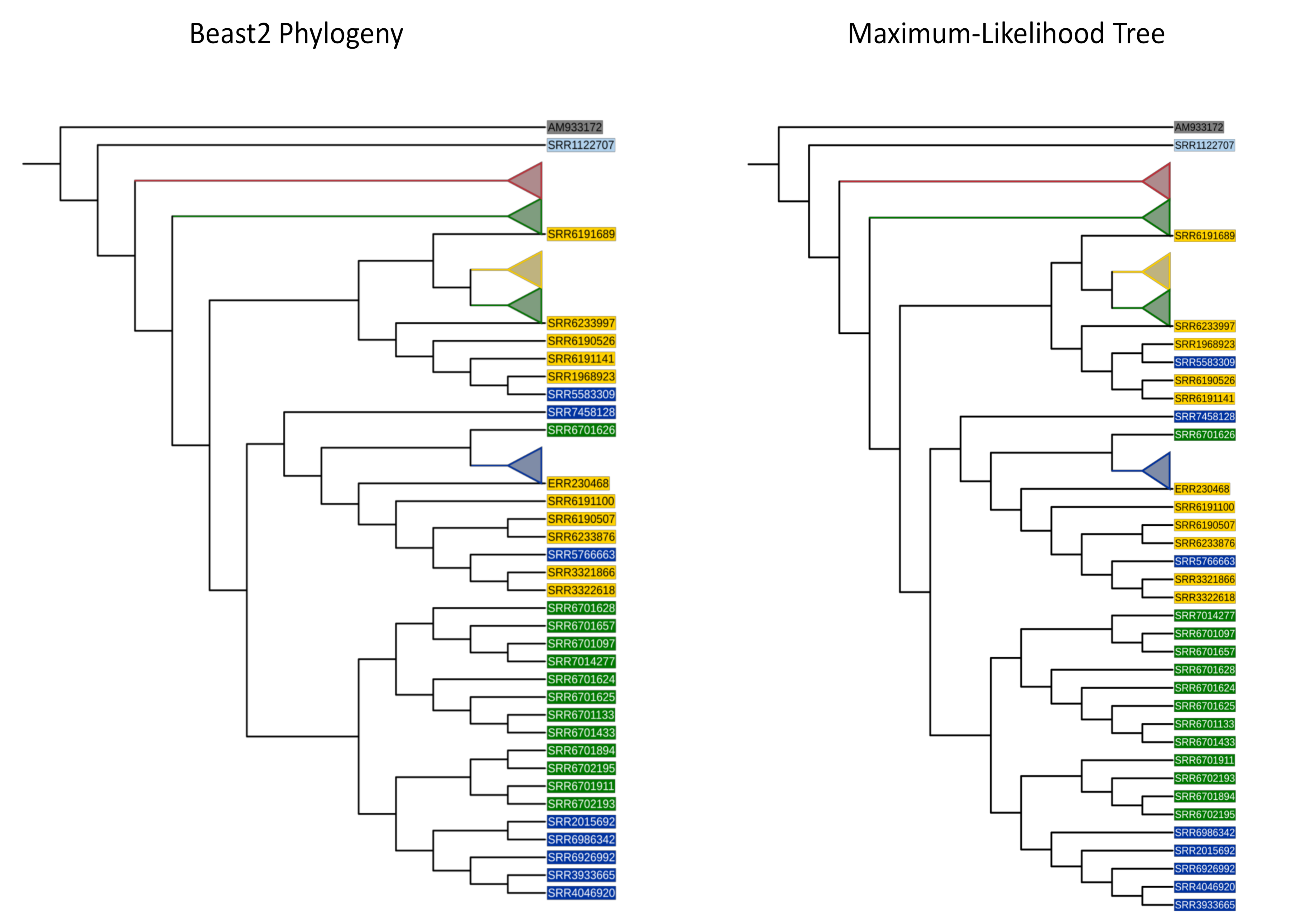

Supplement: evz158_Supplementary_Data [file evz158_supplementary_data.zip › Supplemental_FIG_03.tif]
